# Tissue Culture Study of The Medicinal Plant Leek (Allium Ampeloprasum L)

**Published:** 2014

**Authors:** Mohammad Bagher Monemi, S. Kamal Kazemitabar, Gholamreza Bakhshee Khaniki, Esmaeil Yasari, Firouzeh Sohrevardi, Roghayeh Pourbagher

**Affiliations:** 1 *Department of Agriculture, Payame Noor University, Iran.*; 2 *Sari Agricultural Science and Natural Resource University, Iran.*; 3 *Gabit and Agricultural Biotecnology Institute of Tabarestan, Sari Agricultural Sciences and and Natural Resources University, Iran.*; 4 *Cellular and Molecular Biology Research Center (CMBRC) Babol University of Medical Sciences, Babol, Iran.*

**Keywords:** Leek, Allium Ampeloprasum, tissue culture, chromatography, essential oil

## Abstract

Persian shallot, also called leek (*Allium ampeloprasum*), is a monocotyledon plant of the lily family (Liliaceae). It belongs to the genus Allium, has a characteristic taste and morphological features, making it to be considered as one of the popular herbal medicine. This research was conducted with the purpose of obtaining optimal conditions for tissue culture of Persian shallot and comparing its active ingredient production *in vitro* versus *in vivo*. In this study, the auxin 2, 4–D and benzyl aminopurine- 6 (BAP) hormones, each at two concentrations (0.5 and 0.1 mg/ L) and Kin at 0.5 mg/ L were used in the format of a randomized complete block design in three replications. Results showed that the best culture media for callus formation for leaf and seed explants were the MS cultures with the hormonal compositions (0.5 mg/ L of 2, 4– D, 0.1 mg/ L of BAP) and (0.5 mg/ L of Kin and 0.1 mg/ L of 2, 4– D). Identification of the chemical composition of the essential oils, extracted either from leek callus or leaf was carried out using GC mass analysis. Twenty one compounds were detected in the GC mass spectra, seven of which constitutv about 51.5% of the total amount of compounds present in the essential oils were identified. Our data demonstrate that the leek essential oil constituents as well as callus formation can be affected by culture medium condition.

Leek or great round– headed garlic, perennial sweet leek, blue– wild– vine leek, pearl leek, and summer leek (*Allium ampeloprasum* L.) is one of the widely used vegetables in Iranian diet and is considered as a medicinal plant (1). It belongs to the lily family (Liliaceae), is dioecious and perennial, with band– shaped, relatively broad leaves with long sheaths; and has not been observed as a wild plant yet. The main components in leek essence are pentanol (18%), 5,2-methyl furan (7%), octa decan (9%), dipropyl disulphide (5.6%), methyl alil sulphide (4.3%), tetra hydro 5-2 dimethyl tiophone (4.4%) and kamphore (3.2%). These components in leek may cause intestinal cancer reduction while 2-methyl furan causes blood cholesterol reduction and camphor is also considered as a disinfection component. Different enzymes such as maltase, doctrinase and invertase are available in leaves, as well as important amounts of iron and vitamin C. Vitamin B_1_ and B_2_ can be found in summer. In addition, leek contains manganese, calcium, phosphorus, sodium, potassium, vitamin A and B_6_.

Plant tissue culture technology is very important in agriculture and in industry (2-3). Among the applications of plant tissue culture are mutant plant and haploid production, asexual reproduction, pathogen– free plant production, and production of plants with new genetic information (4-5).

The basis of plant tissue culture was proposed in 1902 by Gottlieb Haberlandt of The German Academy of Sciences. After the experiments, he carried out on propagating unicellular plants (6-10). Silvertand et al. introduced a simple and efficient method for propagating leek in laboratories. They used flower stems and cultured these samples to produce adventitious branches (11). In their research, Mohamed Yasseen and Costanza introduced two different protocols for clonal propagation of a cultivar of leek called Kurrat. They were grown in the MS base culture medium containing different amounts of benzyl aminopurine- 6 (BAP) or naphtyl acetic acid (NAA) hormones. After four weeks, adventitious branches appear. In the two– step method, the explants were first grown in the dark for four weeks in the Murashige and Skoog (MS) medium containing the hormones 2, 4– D. Then, they were transferred to the MS medium containing BA to produce branches. All branches produced in both methods grew roots in the MS medium that contained active charcoal (12-14).

This study was conducted to discover the optimal conditions for tissue culture in leek and investigate the *in vivo* presence of ethereal oils in calli and leaves of leek. Our research can be a basis for investigations on the medicinal properties of this plant or the transfer of genes to this plant.

## Materials and Methods

**Plant materials and culture conditions**

Seeds of local leek (*Allium* spp) were soaked in water for 6 hours and then sterilized in the sterile chamber for 10 mins in a solution containing 1.25 % sodium hypochlorite to which a few drops of 20% tween had been added. Then, the sodium hypochlorite was slowly drained and the seeds were washed five times, each time for two mins, with sterile distilled water. Then, the sterilized seeds were grown in the hormone– free MS medium to produce sterile plant. The sterilized explant samples, including leaf segments obtained from seed culture were evaluated. These seeds had been cultured in the base culture medium MS that contained 30 g/ L of sucrose and 8 g/ L of agar, the hormone kinetin and auxins such as 2, 4– D, kinetin, and BAP at different concentrations. The hormonal treatments using explant– leaf samples used in this study were as follows: the 2, 4–D treatment at two concentrations of 0.1 and 0.5 mg/ L, the Kin treatment at the 0.5 mg/ L, the BAP treatment at two concentrations of 0.1 and 0.5 mg/ L (with 2, 4– D and Kin studied in combination and 2, 4– D and BAP studied separately) in the format of randomized complete block design and in three replications. The leaf explants and seed samples transferred into petri dishes containing 25 ml of the culture medium were placed in the dark at 25± 2^°^C. The percentages of callus formation were measured and the mean callus wet weights were compared to find the best hormonal treatment condition.

**Extraction of essential oils**

Extraction of the essential oils of Allium sp.(leek) was performed using the method of distillation with water and the Clevenger apparatus from 30 grams of callus during 180 mins of extraction time.

The essential oils of leek were extracted using the method of distillation with water and injected into the GC- mass apparatus under thermal scheduling. After injection, the GC-mass spectra and the mass spectra of the constituents of each essential oil were obtained. Then, the constituents of each essential oil, Kovats retention indices (K1), and the percentage of each constituent were determined by comparing the mass spectra of unknown compounds with those of standard compounds, and also by calculating retention indices. The gas chromatograms show the peaks according to the order of retention times. The greater the area under the peak, the higher the percentage of the constituent in the essential oil.

**Chemical analysis**

The essential oils were identified using the chromatography method by GC mass (model Hewelet 6890 with Detector 5975C). After extracting ther by the Clevenger, the essential oil was dried by dry sodium sulfate and then injected into the GC mass under the thermal scheduling program. Next, the constituents of each essential oil were determined.

**Statistical analysis**

The experimental data were evaluated by employing the SPSS software on the basis of Duncan’s multiple range test at 5% probability level.

## Results

**Leaf segments**

The leaf segment explants and the seed samples started to swell gradually after they were cultured in the callus formation medium for two weeks, and started to produce calli after 3 weeks. In most experimental treatments, the majority of leaf explant samples and all seed samples turned into calli. Therefore, the weights of the calli formed in the treatments were compared in order to compare different treatments.

The comparison of means revealed that the effects of different hormonal compounds and their mutual effects on callus formation of leaf explants (Table 1) and seed samples were significant at 1% probability level.

**Effects of the 2, 4– D and BAP on the callus**

Comparison of the means of the data indicated that the hormonal treatments containing 0.5 mg/ L of 2, 4– D and 1 mg/ L of BAP (Table 2), and the hormonal treatments containing 0.1 mg/ L of 2, 4– D and 0.5 mg/ L of Kin (Table 3), had the greatest ability in forming calli. Therefore, these conditions were identified as the best treatments for callus formation.

**The effects of the type of leaf** **explant samples** **on the percentage of callus formation**

The results obtained from analysis of variance of the type of leaf explant samples (Tables 2 and 3) showed that the type of leaf explant had a significant effect on the callus wet weight at 1% probability level. On the basis of Duncan’s multiple range test, it was revealed that the largest callus wet weight was that of the leaf explant samples cultured in 0.25 mg/ L 2, 4– D, and the smallest were those of the leaf explant samples cultured with 0.5 mg/ L of 2, 4– D and 1.0 mg/ L of BAP 

**Table 1:** Analysis of variance of the effect of hormonal compounds on percentage of callus formation in leaf explants and seed samples

**Source of variation**	**degree of freedom**	**MS (leaf)**	**F**	**MS (seed)**	**F**
Treatment	5	3003.855	5.699**	2817405.543	0.018^ns^
Error	12	527.105		2766510.035	
Total	17				

MS (Mean Square), F (Fisher),

** Significant at the 1% level**, **ns (non- significant)

The results concerning the analysis of variance of the effects of different growth regulators on the percentage of callus formation in Allium sp. leaf explants and seeds are presented in Table 1. These results showed that the hormonal treatments had a significant effect on the percentage of callus formation.

**GC- mass spectra**

**Fig 1:**
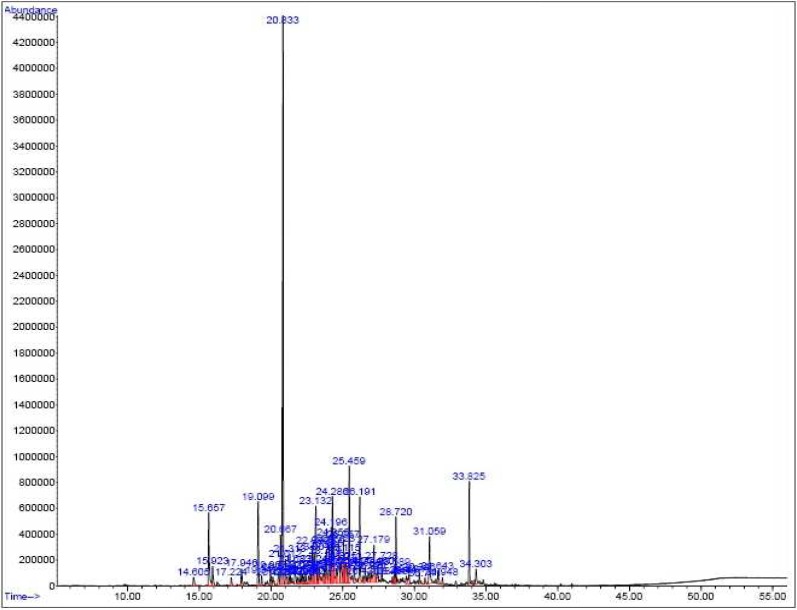
GC- mass spectra of the essential oils from the leaves of *Allium* sp

**Fig 2:**
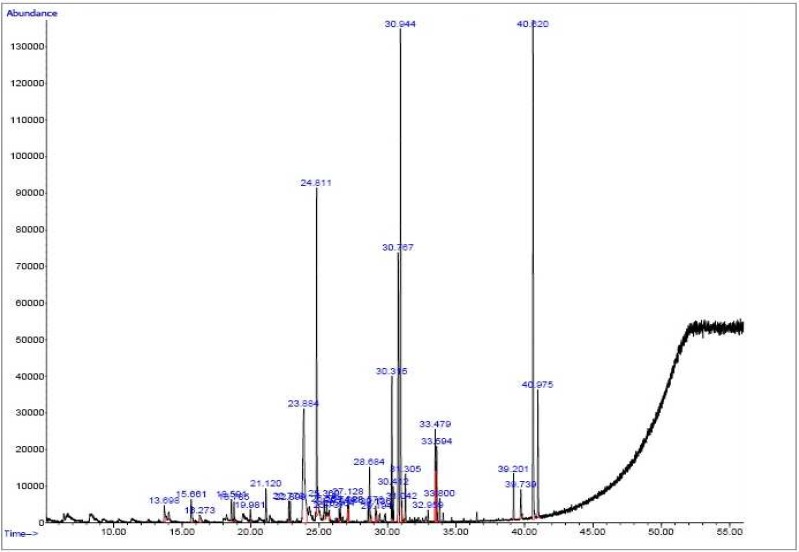
GC- mass spectra of the essential oils of the calli fram the plant *Allium *sp

The percentage of the essential oils (w/w) were 0.3% from callus organ by the water extraction method. The peaks of the GC- mass of the essential oil of the plant leek obtained from leaf culture and the essential oil of its callus obtained by water extraction are presented in Figures 1 and 2 respectively.

**Table 2:** The effects of the hormones 2, 4– D and BAP on the percentage of callus formation

**Type of callus**	**% of callus formation**	**2, 4- D µg/ml**	**BAP µg/ml**
No callus formed	-	0.0	0.0
White callus	+	0.0	0.5
Colorless callus	+	0.1	0.0
Yellow callus	+++	0.1	0.5
Colorless callus	+	0.5	0.0
White callus	+	0.5	0.5

The Symbols +, ++ and +++ mean that the percentages of callus formation are low, average and more than 60, respectively

**Constituents of the essential oils of leaves and calli of leek**

The active constituents in the leaves of leek accounting for 96.5% of compounds found in these leaves (shown in Table 4) were naphthalene, cycloisolongifolene, 3– methyl– 4 isoprophylph-enol, thymol, and caryphyllene, respectively. Moreover, 30 g of wet callus of leek were extracted using the Clevenger apparatus, the extract was injected into the GC/ MS apparatus, and the compounds present in the extract were identified.

Table 5 shows the contents of the active constituents present in the calli produced by culturing leek (which constitute 85.18 % of the total 100 % of the compounds found in the calli) .

**Table 3:** The effects of the hormones 2, 4– D and Kin on percentages of callus formation

2, 4 –D µg/ ml	Kin µg/ ml	Type of callus	Percentage of callus formation
0.0	0.0	No callus formed	-
0.0	0.5	Milk- colored callus	+
0.0	1.0	No callus formed	+
0.5	0.0	Dark reddish callus	+
0.5	0.5	Dark reddish callus	++
0.5	1.0	Dark reddish callus	++
0.5	0.0	Milk– colored callus	+
0.5	0.5	Dark reddish callus	++
0.5	1.0	Green callus	+++

Symbols +, ++ and +++ mean that the percentages of callus formation are low, average and more than 60, respectively

**Table 4:** The quantities of active constituents present in the leaves of leek

Estimated percentage	Active constituent	% area	R.T
91	Bicyclo, hept– 2– ene	0.56	14.605
90	Thymol	3.68	15.655
97	3– methyl– 4 isoprophylphenol	3.68	15.924
99	Caryphyllene	3.63	19.099
99	Naphthalene	23.27	20.833
64	Copaene	2.96	24.268
55	Cycloisolongifolene	4.76	25.459
93	2– pentadecanone	1.96	28.720
98	N– Hexadecanoic acid	2.49	31.059
86	Phytol	3.16	33.825
99	9, 12, 15– octadecatrienoic acid	0.81	34.303
Total		50.96	

RT: Retention Time

**Table 5:** The quantities of active constituents present in the calli of leek

**Estimated percentage**	**Active constituent**	**% Area**	**R.T**
83	Cyclotetrasiloxane	1	13.69
80	Phenol, 2 methyl 5	1.44	15.661
37	1, 1–Bis (difluoromethyl) ethyl iso	1.26	21.20
59	Ethyl. alpha– d- glucopyranoside	13.25	23.884
47	2– methylindene	11.64	24.881
62	9.methyl– 3.4– dihidro– 2h– pyrido	2.11	28.684
97	Pentadecanoic acid	4.11	30.315
52	2H– 1– Benzopyran,	9.19	30.769
58	3.4– dihidro & H– Cyclopenta	16	30.944
99	9.12. octadecadienoic acid	2.43	33.479
10	1.3.5, cycloheptatriene	1.20	39.201
12	3.morpholione.5– methyl– 6.phenyle	1.18	39.739
27	Benzene	16.04	40.620
86	1, 2. Benzenedicarboxyl	4.33	40.975
Total	-	85.18	

RT: Retention Time

## Discussion

Several endogenous and exogenous factors on the plant are effective in callus formation and growth. The chemical factors, mineral elements, and growth regulators are the most important growth factors that influence plant differentiation and growth initiation. In cell culture, growth and morphogenesis are similarly controlled by the type and concentration of plant hormones and the mutual relationships between the hormones. In our study, an auxin and a cytokinin were used as hormones to induce callus formation. Auxins are commonly required to induce callus formation in leaf explants and seed samples. The most common auxins used for inducing callus formation is 2, 4–D. However, the use of 2, 4– D must be limited as much as possible, because it can cause mutation and may also prevent photosynthesis. Moreover, The joint use of cytokinins and auxins results in the stimulation of cell division. In this research, the hormones 2, 4– D, BAP, and Kin were used to induce callus formation. In some of the treatments, callus formation was induced due to the presence of auxins; and this shows the importance of the use of auxins in inducing callus formation. In our study, it was found that the mutual effects of these three hormones were significant, and that the highest percentage of callus formation belonged to the T4 and T5 treatments, which included the use of 0.5 mg/ L of 2, 4– D and 1 mg/ L of BAP, and 0.1 mg/ L of 2, 4– D and 0.5 mg/ L of Kin, respectively. The hormone 2, 4– D at the three levels of 0.5, 0.1, and 0.0 mg/ L, and the hormone Kin at the two levels of 0.5 and 0.0 mg/ L were used in separate treatments to evaluate their effects. Comparison of the appearances of the calli obtained in media containing different hormonal combinations showed that in media including 2, 4– D as the only source of inducing callus formation the calli were white, while yellow calli were produced in media containing BAP. The type of leaf explants sample is one of the important factors that greatly influence callus formation. In this experiment, which was conducted to compare the effects of the type of explants sample on the percentage of callus formation, the largest callus wet weight (1.3020 g) belonged to explants leaf sample (as compared to seed samples).

These results show that the hormonal treatments have not had any significant effect on callus formation. The embryogenic callus cultures were friable, nodular and similar in appearance to those obtained leek (15-16). The preferred callus type was often surrounded by soft watery callus; therefore, making sustained selection for the regeneration callus type is essential. Immature embryos have been successfully used to initiate embryogenic callus cultures for the major species inside the genus *Allium *(13, 15, 17-18).

The active constituents of essential oil according to table 5, were benzene, 7 H– cyclopenta, ethyl alpha– d- glucopyranoside, 2– methylindene, 2H– 1– benzopyran, 3.4– dihidro, respectively, and they were found in the largest quantities. Tables 4 and 5 indicate the percentage of different constituents produced in calli and the leaves are different although their origin unique.

The obtained results indicate that the leek essential oil constituents as well as callus formation can be affected by culture medium condition. Since the active ingredients of leek have many pharmacological properties, by controlling the culture medium ingredients, the quality and constituents of leek essential oil produced *in vitro* might be manipulated in a way to optimize the bioactive molecules production toward useful medicinal drugs.

## Conflict of interest

The authors declared no conflict of interest.
